# An Exploratory Analysis of Rhythmic Auditory Stimulation's Impact on Brain Function in Parkinson's Disease Patients With Freezing of Gait

**DOI:** 10.1002/brb3.70532

**Published:** 2025-05-08

**Authors:** Chen Liu, Miaomiao Hou, Shuili Yu, Ying Wan, Zhenguo Liu, Jing Gan

**Affiliations:** ^1^ Department of Neurology Xinhua Hospital Affiliated to Shanghai Jiaotong University School of Medicine Shanghai China

**Keywords:** fNIRS, freezing of gait, Parkinson's disease, rhythmic auditory stimulation

## Abstract

**Introduction:**

Freezing of gait (FOG) is a debilitating symptom in Parkinson's disease (PD) patients that severely impairs mobility. Rhythmic auditory stimulation (RAS) has demonstrated potential in improving gait, but the mechanisms underlying its efficacy remain unclear. This study explored the impact of RAS on brain function in PD patients with FOG using functional near‐infrared spectroscopy (fNIRS) during an imagined walking task, with the aim of elucidating the underlying mechanisms involved.

**Methods:**

We enrolled 45 PD patients, comprising 21 with FOG (PD+FOG), 24 without FOG (PD‐FOG), and 10 healthy controls (HC). Using a 53‐channel fNIRS system, we measured the oxygenated hemoglobin (oxy‐Hb) content in brain regions as subjects performed an imagined walking task both with and without RAS. Dynamic functional connectivity and k‐means clustering were employed to identify key brain connectivity states. Data processing was conducted using MATLAB R2014a, with one‐way ANOVA to select channels of interest, followed by paired t‐tests for comparative analysis.

**Results:**

PD+FOG patients showed significantly lower activation in the supplementary motor area (SMA) and the left primary motor cortex of the lower limb (M1‐LL) without RAS compared to PD‐FOG patients and HC. RAS significantly increased activation in these areas and altered functional connectivity, characterized by an increased frequency of transitions between low‐strength and medium‐strength connectivity states in PD+FOG patients.

**Conclusion:**

RAS may ameliorate gait in FOG patients by activating key motor areas (SMA and left M1‐LL) and modulating the dynamic regulation of brain connectivity. This research provides valuable insights into the neural processes involved in the therapeutic effects of RAS.

## Graphical abstract

Rhythmic auditory stimulation may ameliorate freezing of gait through two potential mechanisms: (1) activating the supplementary motor area and the left primary motor cortex of the lower limb and (2) reducing the stability of brain connectivity states, through which modulating the dynamic regulation of brain connectivity.

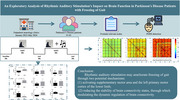

## 1 Introduction

Freezing of gait (FOG) is a debilitating symptom that significantly impacts individuals with Parkinson's disease (PD). In China, approximately 30% of PD patients experience FOG, with this prevalence increasing as the disease progresses (Gan et al. 2021). FOG is characterized by “a brief, episodic absence or marked reduction of forward progression of the feet despite the intention to walk” (Giladi and Nieuwboer 2008), during which patients often describe the sensation as if their feet are “suddenly glued to the ground.” It typically occurs during activities such as initiating movement, turning, navigating narrow spaces, or approaching a destination (e.g., a chair), resulting in an elevated risk of falls and reduced independence, which severely compromises the quality of life of PD patients (Mirelman et al. 2019).

Rhythmic auditory stimulation (RAS) is a neuromusical therapy technique commonly employed in gait training, involving regular, isochronous auditory pulses. Studies have shown that RAS is a safe, cost‐effective, and beneficial approach for enhancing gait in PD patients (Ashoori et al. 2015). Specifically, RAS has been found to reduce both the frequency and duration of freezing episodes, particularly during activities such as walking in a straight line, turning, and navigating obstacles (Devlin et al. 2019). Additionally, RAS improves stride speed, stride length, and gait rhythm (Trindade and Viana 2021).

Despite numerous hypotheses and models proposed to explain the pathophysiological mechanisms underlying FOG, most focus on cognitive executive dysfunction and visuospatial processing abnormalities within cortical and subcortical signaling pathways. The “interference model,” “executive dysfunction model,” and “perceptual dysfunction model” emphasize impaired executive control and perception as contributing factors to the motor symptoms observed in FOG (Bardakan et al. 2022). However, these models often neglect the role of the auditory signaling system, particularly with respect to rhythmic cues. As a result, the exact neural mechanisms through which RAS alleviates FOG remain unclear. Recent studies suggest that neural activity associated with rhythm perception is closely linked to motor regulation, involving key cortical and subcortical regions such as the premotor cortex, supplementary motor area (SMA), cerebellum, and basal ganglia (Raglio 2015). One prominent hypothesis is that RAS may facilitate the synchronization of neural circuits involved in motor control by enhancing the coupling between auditory and motor regions. Specifically, auditory rhythm may help entrain motor patterns, thereby improving gait performance. Additionally, RAS may activate subcortical structures like the basal ganglia, which are essential for motor initiation and coordination (Grahn and Brett 2007). This involvement of neural circuits during rhythm perception offers a promising avenue for understanding how RAS may help alleviate FOG symptoms.

The functional magnetic resonance imaging (fMRI) studies have indicated that passive listening to rhythmic stimuli activates both auditory regions and SMA neurons, even without explicit motor action (Chen et al. 2008). This suggests that RAS might facilitate motor planning by engaging the SMA, which, in turn, could influence the primary motor cortex of the lower limb (M1‐LL) to enhance motor execution during walking. Building upon this, we propose an expansion of the “perceptual dysfunction model” by incorporating auditory perception as a crucial component of sensory processing. We hypothesize that auditory stimuli can activate sensory pathways involved in rhythm perception, which subsequently affect brain regions responsible for motor control—specifically, the SMA. The SMA plays an essential role in motor planning and coordination. From the SMA, motor commands are transmitted to the primary motor cortex (M1), particularly its lower‐limb area (M1‐LL), responsible for executing walking movements. This cascade of neural activation, from auditory perception through the SMA to M1‐LL, offers a promising framework for understanding how RAS may alleviate FOG symptoms.

To investigate these neural mechanisms, functional near‐infrared spectroscopy (fNIRS) emerges as a valuable tool. This non‐invasive optical brain imaging technique offers a compromise between the spatial resolution of fMRI and the temporal resolution of electroencephalography (EEG), allowing for real‐time monitoring of cerebral hemodynamics and functional cortical activity (Huo et al. 2024). Additionally, motor imagery (MI)—the mental visualization of specific movements—activates brain regions involved in movement planning, similar to actual movement execution (Hétu et al. 2013). Thus, MI has become a key paradigm for investigating brain functions related to FOG.

The present study aims to employ the MI research paradigm in conjunction with fNIRS to explore differences in cortical activation and dynamic functional connectivity between PD patients with FOG (PD+FOG) with and without RAS. These findings will be compared with those of PD patients without FOG (PD‐FOG) and healthy controls. The goal is to uncover the potential mechanisms through which RAS improves FOG and gain further insights into its neural effects on motor control.

## 2 Materials and Methods

### 2.1 Subject Selection

Between January 2023 and May 2024, 45 idiopathic PD subjects were enrolled from outpatient clinics of neurology departments of Xinhua Hospital affiliated with Shanghai Jiao Tong University School of Medicine (XH‐SJTUSM). Inclusion criteria: (1) diagnosed with idiopathic PD by an experienced neurologist according to 2015 Movement Disorder Society Clinical Diagnostic Criteria (Postuma et al. 2015). (2) Stable medication regimen for at least 1 month prior to recruitment. (3) Age ≥ 50. (4) Hoehn‐Yahr(H‐Y)stage I‐III. (5) Can cooperate with evaluation and physical examination. Exclusion criteria: (1) patients with secondary Parkinsonism, Parkinsonism‐plus syndrome, or other pre‐existing neurological disorders (such as epilepsy, encephalitis, etc.). (2) Have a history of deep brain stimulation surgery. (3) Have an obvious head tremor. (4) Have severe psychiatric symptoms and cardiopulmonary or other internal diseases. (5) The Mini‐Mental State Examination (MMSE) (Folstein et al. 1975) score < 24 points.

The healthy control group (HC) consists of family members accompanying PD patients for their appointments, elderly individuals visiting the outpatient clinic for check‐ups at our hospital during the same period, and older adults from nearby communities. Inclusion criteria: (1) No clear neurological diseases and no significant internal medical conditions. (2) The MMSE score >24 points.

Subjects were thoroughly briefed on the study's objectives and methods, and they provided written informed consent before their involvement. The study was approved by the Ethical Committee of Xinhua Hospital affiliated with Shanghai Jiao Tong University School of Medicine (Approval No.: XHEC‐C‐2015‐019‐2).

### 2.2 Clinical Assessments

All PD patients underwent clinical assessments and fNIRS examinations in an ON‐medication state (one hour after taking their regular antiparkinsonian medication and having a good therapeutic effect). Demographic information, including age, gender, educational level, age of PD onset, duration of the disease, and medication status, was gathered through a comprehensive interview. The levodopa equivalent daily dose (LEDD) (Tomlinson et al. 2010) was calculated accordingly. To evaluate the severity of motor symptoms, the H‐Y stage and Movement Disorder Society‐Unified Parkinson's Disease Rating Scale Part III (MDS‐UPDRS III) (Goetz et al. 2008) scores were utilized. The New Freezing of Gait Questionnaire (NFoGQ) (Nieuwboer et al. 2009) was used to assess freezing of gait, and based on the scores from the first part of the NFoGQ, PD patients were divided into two groups: a PD+FOG group with a score ≥ 1 and a PD‐FOG group with a score = 0. The Frontal Assessment Battery (FAB) (Dubois et al. 2000), MMSE, and Montreal Cognitive Assessment (MoCA) (Rossetti et al. 2011) were used to evaluate cognitive function.

Record the age, gender, and educational level of subjects in the HC group, and assess their cognitive function using the FAB, MMSE, and MoCA scales.

### 2.3 fNIRS Examinations

**FIGURE 1:**
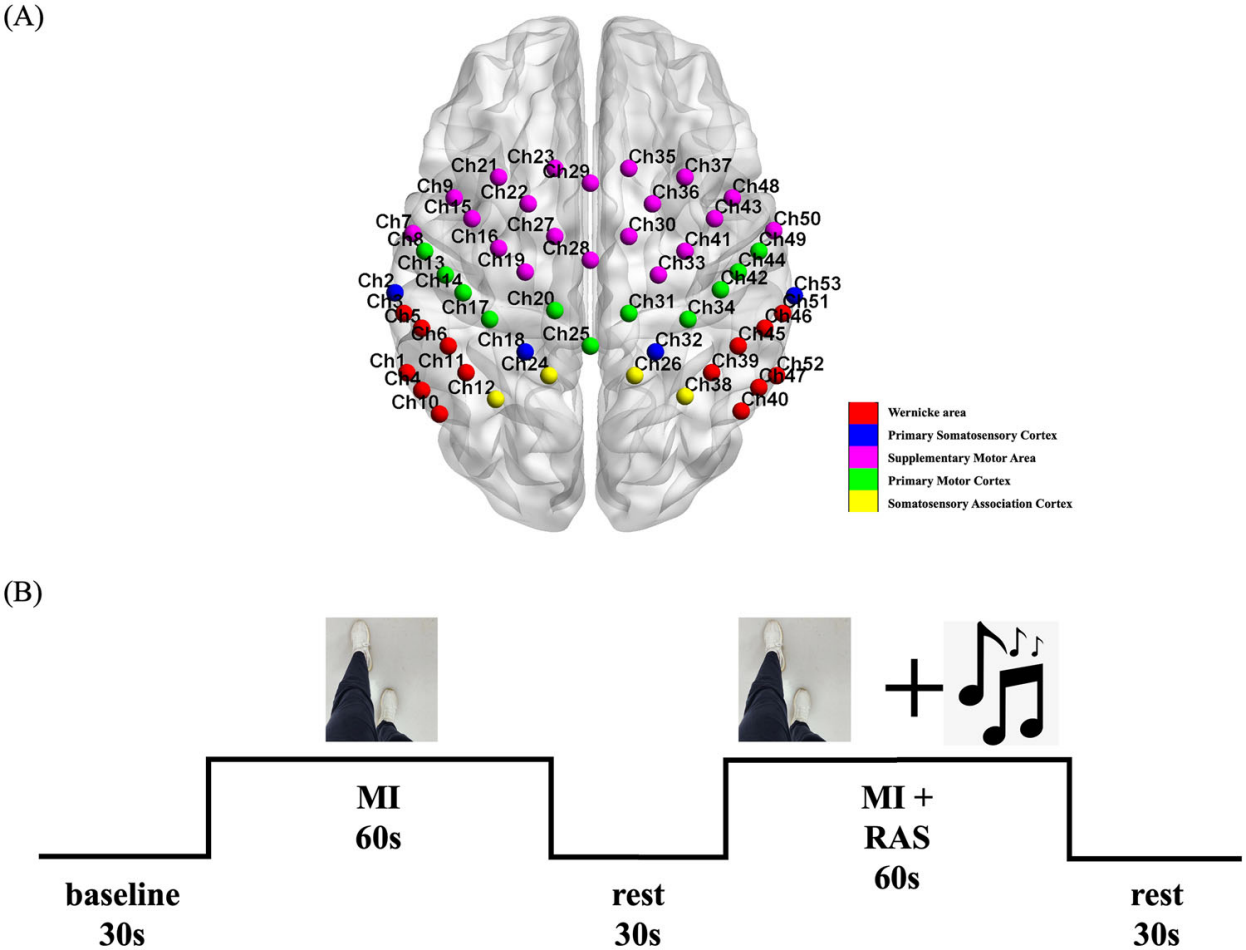
(A) The arrangement of channels of the 53‐channel near‐infrared spectroscopy system according to the Brodmann's map of the cortex. (B) Flowchart of the task procedure in this study. MI, motor imagery; MI + RAS, motor imagery with rhythmic auditory stimulation.

This study used the BS‐7000 fNIRS (Wuhan Znion Technology Co., Wuhan, China) to continuously collect the concentration of oxygenated hemoglobin (oxy‐Hb) in the cerebral cortex to understand its neural activity and hemodynamic responses. The device employs 53 measurement channels at a 20 Hz sampling rate, using two near‐infrared light sources at 690 nm and 830 nm wavelengths to evaluate oxyhemoglobin (oxy‐Hb), deoxyhemoglobin (deoxy‐Hb), and total hemoglobin (Total‐Hb) concentrations in the cerebral cortex. A head cap equipped with near‐infrared light sensors covered the frontal, parietal, and temporal lobes of the subjects. Distribution of near‐infrared sensors adhered to the international standard of 10–20 leads, with Channel 28 positioned at Cz. Measurement regions included the bilateral SMA (Ch7, Ch9, Ch15, Ch16, Ch19, Ch21, Ch22, Ch23, Ch27, Ch28, Ch29, Ch30, Ch33, Ch35, Ch36, Ch37, Ch41, Ch43, Ch48, Ch50), the M1 (Ch8, Ch13, Ch14, Ch17, Ch20, Ch25, Ch31, Ch34, Ch42, Ch44, Ch49), the primary somatosensory cortex (S1) (Ch2, Ch18, Ch32, Ch53), Wernicke's area (Ch1, Ch3, Ch4, Ch5, Ch6, Ch10, Ch11, Ch39, Ch40, Ch45, Ch46, Ch47, Ch51, Ch52), and the somatosensory association cortex (SAC) (Ch12, Ch24, Ch26, Ch38), as delineated by the Brodmann map of the cortex (Figure 1A).

The experimental process required a quiet environment with a suitable temperature, where the subjects were relaxed and seated comfortably in a chair. Once the head cap was securely positioned, subjects were required to look at the computer screen in front of them, taking care to avoid any movement of the head. Prior to the start of the experiment, the master experimenter, who was unaware of the subjects' conditions and group assignments, provided a standardized explanation of the experimental tasks and precautions. Once it was confirmed that the subjects could correctly complete the tasks, the formal experiment commenced.

### 2.4 Motor Imagery Tasks

This study was designed based on an fMRI study using the MI paradigm (Huang et al. 2021), employing the “first‐person‐perspective” video clips to investigate changes in cortical activation levels under different task conditions. As shown in Figure 1B, this study included two tasks, each with a duration of 60 seconds. (1) Task 1 (no‐RAS condition): a video was displayed on the computer screen, showing a pair of legs walking forward at a normal pace for a duration of 20 seconds before stopping. The video was repeated three times, and subjects were instructed to watch the video and imagine themselves walking during the process. (2) Task 2 (RAS condition): the same video was displayed on the computer screen, repeated three times. While the subjects watched the video, a rhythmic piece of music was played simultaneously. The walking rhythm in the video was synchronized with the rhythm of the music. The subjects were instructed to watch the video and imagine themselves walking in sync with the music.

After completing both tasks, participants were given a 30‐second rest period. During this time, numbers from 1 to 15 were displayed sequentially on the screen. Participants were asked to read the numbers aloud according to the on‐screen prompts, with the volume adjusted to a level audible to the operator. This rest period was designed so as to ensure consistent attention and cognitive load across participants throughout the experiment. Specifically, the number reading task served as a simple, low‐demand activity to control for potential confounding variables, allowing for a clear comparison with the more cognitively demanding imagery walking tasks.

### 2.5 Statistical Analysis

Results for normally distributed continuous data were presented as mean ± standard deviation($\bar{x}\pm s$). For non‐normally distributed continuous data, values were reported as median (interquartile range) [M (Q1, Q3)]. Categorical data were summarized as counts. The comparisons of age, educational level, FAB scores, and MoCA scores among groups were performed using one‐way ANOVA. The comparisons of disease duration, LEDD, and UPDRS‐III scores between groups were conducted using independent samples t‐tests. The comparison of H‐Y staging between groups was analyzed using the rank‐sum test. The comparison of gender among groups was performed using the chi‐square (χ^2^) test. P‐values less than 0.05 were statistically significant. All statistical analyses were conducted using SPSS v25.0.

### 2.6 fNIRS Data Analysis

All fNIRS data of brain networks were preprocessed in MATLAB R2014a using the Homer2_UI package. The preprocessing steps included: (1) hmrIntensity2OD converted light intensity to optical density; (2) hmrMotionArtifactByChannel and hmrMotionCorrectSpline identified and corrected motion artifacts; (3) hmrBandPassFilt (0.0–0.1 Hz) removed irrelevant low‐ and high‐frequency components; (4) hmrOD2Conc converted optical density to changes in oxy‐Hb concentration; (5) hmrBlockAvg (‐5–85s) selected and processed task conditions and features. Following preprocessing, a general linear model (GLM) was used in NIRS‐KIT (Hou et al. 2021) to evaluate the changes in oxy‐Hb for each channel of each subject, described in detail in Supplementary Material 1. After calculating the β values in MATLAB R2014a, a one‐sample t‐test was conducted to analyze the β values for each group under the conditions of without RAS and with RAS during imaged walking, resulting in activation maps for the brain regions of each group. Additionally, a one‐way ANOVA was performed to compare the differences in brain region activation among the three groups. The LSD post hoc tests were used for pairwise comparisons of brain regions with statistically significant differences among three groups without RAS. The channels identified as having significant differences in this analysis were defined as the regions of interest (ROIs) for this study. Paired samples t‐tests were then conducted to compare the changes in β values of each group's ROIs during imaged walking with and without RAS. We used a toolbox named BrainNet Viewer to visualize brain activation (Xia et al. 2013). P‐values below 0.05 were deemed statistically significant.

Dynamic functional connectivity refers to the temporal fluctuations of brain functional networks over brief intervals, offering essential insights into the characteristics of these networks (Lu et al. 2023). In MATLAB R2014a, we derived key brain connectivity states using dynamic functional connectivity and k‐means clustering. To construct the dynamic functional connectivity, we employed a sliding‐window correlation analysis. Subsequently, k‐means clustering was employed to pinpoint the key brain connectivity states. The variations within brain functional networks were quantified using dynamic state features, specifically the state occurrence probability and state transition percentage. The specific analysis steps were referenced from the article by Jiewei Lu et al.

## 3 Results

### 3.1 Demographic and Clinical Features

This study encompassed a total of 45 PD patients, including 21 diagnosed with PD+FOG, 24 with PD‐FOG, and 10 HC. As summarized in Table 1, there were no significant differences among the three groups regarding gender, age, educational level, FAB scores, or MoCA scores. However, the PD+FOG group demonstrated statistically significant differences in disease duration, LEDD, and H‐Y staging compared to the PD‐FOG group, with the PD+FOG patients exhibiting longer disease duration, higher LEDD, and more advanced H‐Y stages.

**TABLE 1:** Comparison of general and clinical data among PD+FOG group, PD‐FOG group and HC group.

	PD+FOG (N = 21)	PD‐FOG (N = 24)	HC (N = 10)	Statistical value	P‐value
Gender (male/female)	12/9	13/11	4/6	0.834^a^	0.659
Age (years)	71.3±6.5	69.4±6.4	68.6±8.4	0.678^b^	0.512
Educational level (years)	10.9±2.2	11.4±2.3	11.1±4.0	0.212^b^	0.809
Disease duration (years)	7.5±3.0	5.0±4.7	/	2.141^c^	**0.038**
LEDD (mg)	734.9±210.5	496.2±261.9	/	3.336^c^	**0.002**
UPDRS‐III (on)	33.9±11.3	28.3±9.5	/	1.799^c^	0.079
H‐Y stage (on)	2.0(2.0,3.0)	2.0(2.0, 2.0)	/	−2.659^d^	**0.008**
NFoGQ	23.2±4.3	/	/	/	/
FAB	15.9±2.0	16.3±2.4	16.8±1.9	0.675^b^	0.514
MoCA	22.9±2.4	23.3±2.9	24.4±3.6	1.003^b^	0.374

*Note*: Data are presented as mean ± standard deviation, median (interquartile range) for continuous variables, or frequencies for categorical ones. ^a^ is the χ^2^ value from the chi‐square test, ^b^ is the F value from the one‐way ANOVA, ^c^ is the t value from the two independent samples t‐test, and ^d^ is the U value from the Wilcoxon rank sum test.

Abbreviations: FAB, the frontal assessment battery; HC, healthy controls; H‐Y, Hoehn‐Yahr; LEDD, levodopa equivalent daily dose; MoCA, Montreal Cognitive Assessment.; NFoGQ, the New freezing of fait Questionnaire; PD+FOG, Parkinson's disease patients with freezing of fait; PD‐FOG, Parkinson's disease patients without freezing of fait; UPDRS‐III, Movement Disorder Society‐Unified Parkinson's Disease Rating Scale Part III.

### 3.2 The Cortical Activation Patterns

#### 3.2.1 Activated Channels During Imagined Walking Without RAS for Each Group

**FIGURE 2:**
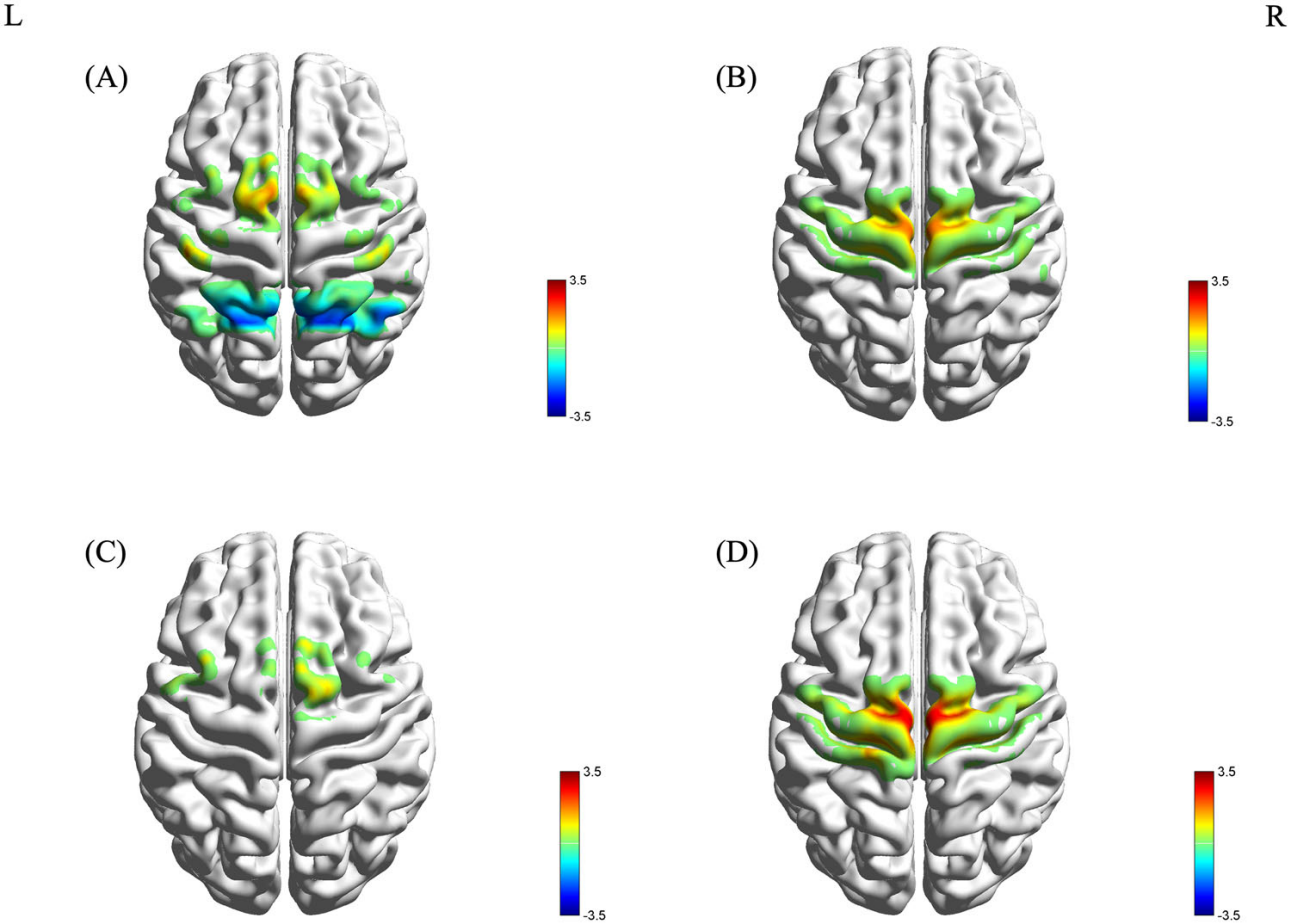
During imagined walking without RAS, the activation of brain regions for the PD+FOG group (2A), PD‐FOG group (2B), and HC group (2C) is presented, along with the distribution map of brain regions showing differences across the three groups from the one‐way ANOVA (2D) (red indicates activation, blue indicates deactivation; L: left, R: right).

In the PD+FOG group, activation was observed in channels Ch14 and Ch42 in the M1, and Ch23, Ch27, and Ch29 in the SMA. Deactivation was noted in channels Ch12 and Ch38 in the SAC, and Ch40 and Ch47 in the Wernicke area (Figure 2A). The PD‐FOG group showed activation in Ch28 (SMA) and Ch46 in the Wernicke area (Figure 2B). The HC group exhibited activation in Ch9, Ch30, and Ch35 in the SMA (Figure 2C).

One‐way ANOVA revealed significant differences in the β values of channels Ch20 and Ch28 across the three groups (F(2, 52) = 3.547, p = 0.037 FDR corrected; F(2, 52) = 4.011, p = 0.024 FDR corrected). LSD post hoc tests indicated that the β values for Ch20 and Ch28 were significantly lower in the PD+FOG group compared to the PD‐FOG and HC groups (Figure 2D). No significant differences were found between the PD‐FOG and HC groups.

#### 3.2.2 Activated Channels During Imagined Walking With RAS for Each Group

**FIGURE 3:**
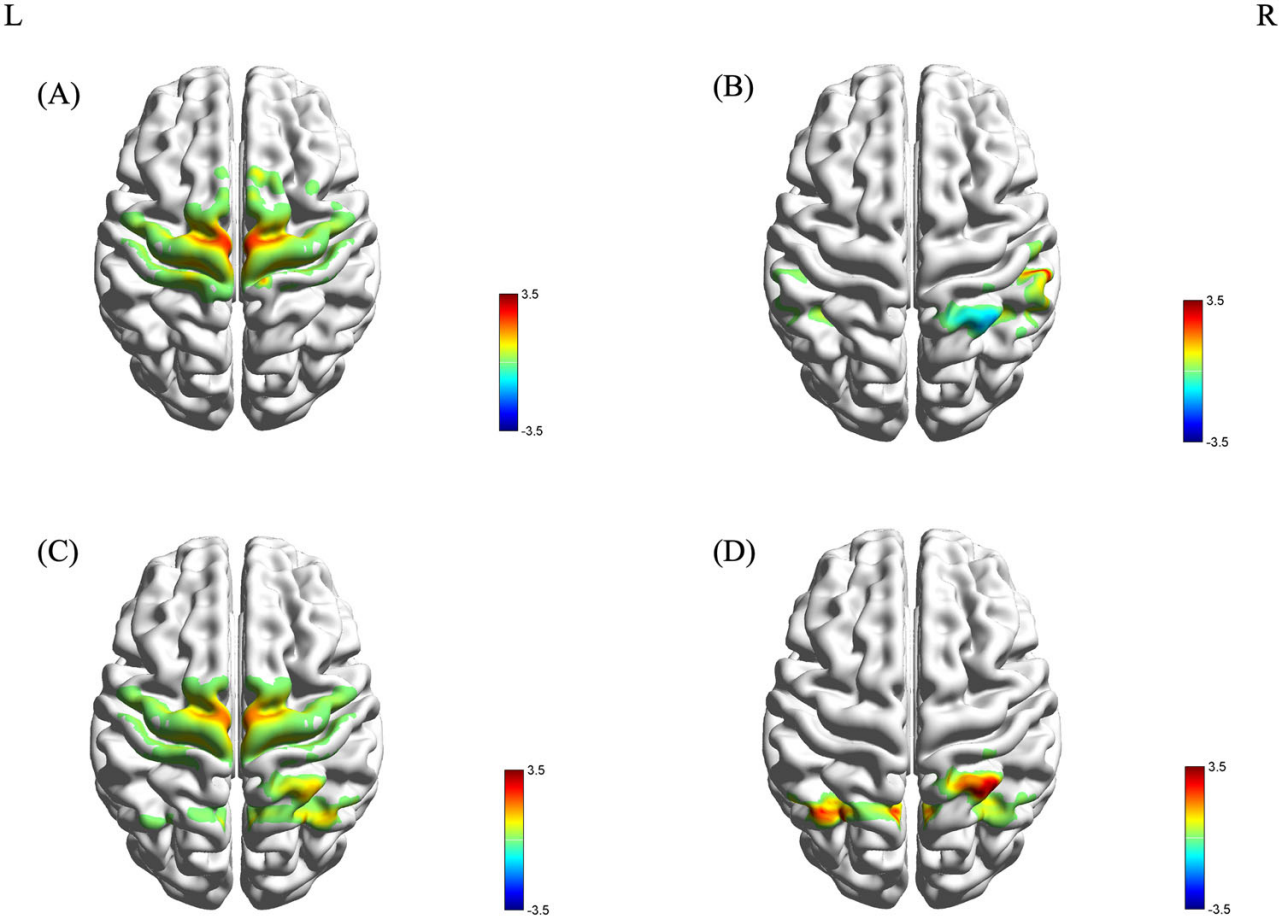
During imagined walking with RAS, the activation of brain regions for the PD+FOG group (3A), PD‐FOG group (3B), and HC group (3C) is presented, along with the distribution map of brain regions showing differences across the three groups from the one‐way ANOVA (3D) (red indicates activation, blue indicates deactivation; L: left, R: right).

During imagined walking with RAS, the PD+FOG group showed activation in Ch28 and Ch35 (SMA), and Ch20 and Ch31 (M1) (Figure 3A). The PD‐FOG group exhibited activation in Ch53 (S1) and deactivation in Ch39 (Wernicke area) (Figure 3B). The HC group showed activation in Ch28 (SMA), Ch39, and Ch40 (Wernicke area) (Figure 3C).

One‐way ANOVA identified significant differences in the β values of channels Ch10 and Ch39 among the groups (F(2, 52) = 4.209, p = 0.021; F(2, 52) = 6.062, p = 0.004). LSD post hoc tests revealed that the β value of Ch10 was significantly higher in the PD+FOG group than in the PD‐FOG and HC groups, while the β value of Ch39 was significantly lower (Figure 3D). There was no difference in β values between the PD‐FOG group and the HC group.

#### 3.2.3 The Changes in the β Values of ROIs During Imagined Walking With and Without RAS for Each Group

Channels with significant differences identified in the initial analysis were defined as regions of interest (ROIs), including Ch20 and Ch28. Further comparisons were made on the changes in β values of each group for imagined walking under RAS and no‐RAS conditions.

**FIGURE 4:**
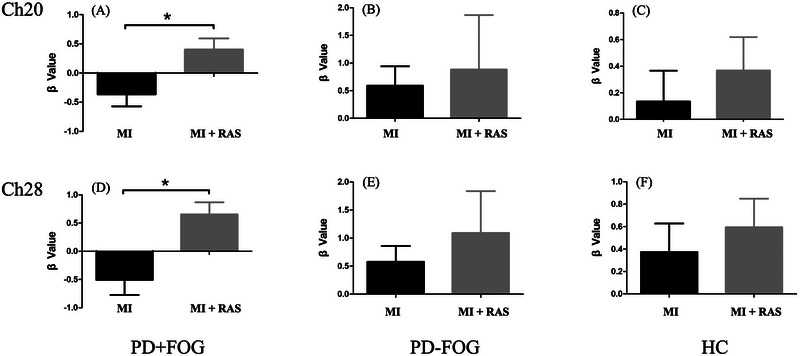
The changes in β values for Ch20 and Ch28 under “MI” and “MI + RAS” conditions in the PD+FOG group, PD‐FOG group, and HC group. MI, motor imagery; MI + RAS, motor imagery with rhythmic auditory stimulation. PD+FOG, Parkinson's disease patients with freezing of gait; PD‐FOG, Parkinson's disease patients without freezing of gait; HC, healthy controls. ∗ indicates p < 0.05.

During imagined walking with RAS, the β value of Ch20 in the PD+FOG group was significantly higher compared to the no‐RAS condition (p = 0.01; Figure 4A, Table 2). Similarly, the β value of Ch28 also showed a significant increase in the RAS condition compared to the no‐RAS condition (p = 0.002; Figure 4D, Table 2). In contrast, the β values of Ch20 and Ch28 in the PD‐FOG group and HC group did not show statistically significant differences between the RAS and no‐RAS conditions (p > 0.05; Figures 4B, 4C, 4E, 4F, Table 2).

**TABLE 2:** The changes in ROIs β values for each group under RAS and no‐RAS conditions($\bar{x}\pm s$).

ROI	group	no‐RAS	RAS	T‐value	P‐value
Ch20	PD+FOG	−0.4±1.0	0.4±0.9	−2.819	**0.010**
	PD‐FOG	0.6±1.5	0.9±4.2	−0.326	0.748
	HC	0.1±0.7	0.4±0.7	−0.545	0.601
Ch28	PD+FOG	−0.5±1.2	0.7±1.0	−3.656	**0.002**
	PD‐FOG	0.6±1.2	1.1±3.1	−0.853	0.406
	HC	0.4±0.8	0.6±0.8	−0.458	0.658

*Note*: The T‐value denotes the statistical value of the β values.

Abbreviations: HC, healthy controls; PD+FOG, Parkinson's disease patients with freezing of gait; PD‐FOG, Parkinson's disease patients without freezing of gait; RAS, rhythmic auditory stimulation; ROI, region of interest.

The results indicated that, during imagined walking with RAS, the activation of Ch20 (left primary motor cortex of the lower leg, M1‐LL) and Ch28 (SMA) in the PD+FOG group was significantly greater compared to the no‐RAS condition.

### 3.3 Dynamic Brain Functional Connectivity States

**FIGURE 5:**
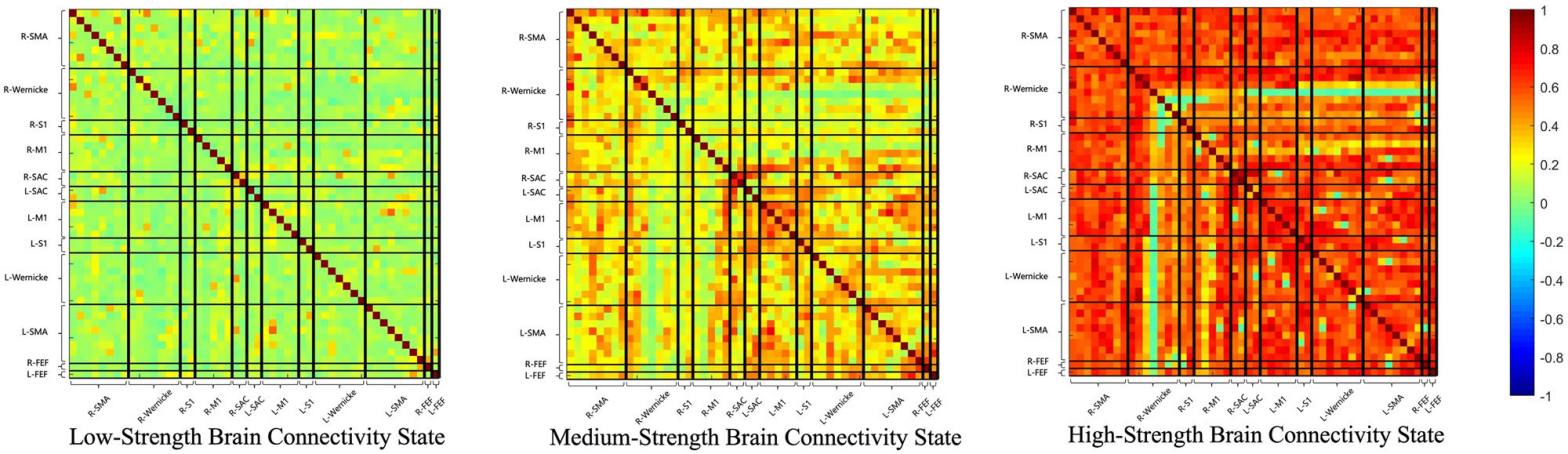
Three key brain connectivity states, named low‐strength, medium‐strength, and high‐strength brain connectivity states. R‐SMA, L‐SMA, R‐Wernicke, L‐Wernicke, R‐S1, L‐S1, R‐M1, L‐M1, R‐SAC, L‐SAC, R‐FEF, and L‐FEF represent the right supplementary motor area, left supplementary motor area, right Wernicke area, left Wernicke area, right primary somatosensory cortex, left primary somatosensory cortex, right primary motor cortex, left primary motor cortex, right somatosensory association cortex, left somatosensory association cortex, right frontal eye field, and left frontal eye field, respectively.

**FIGURE 6:**
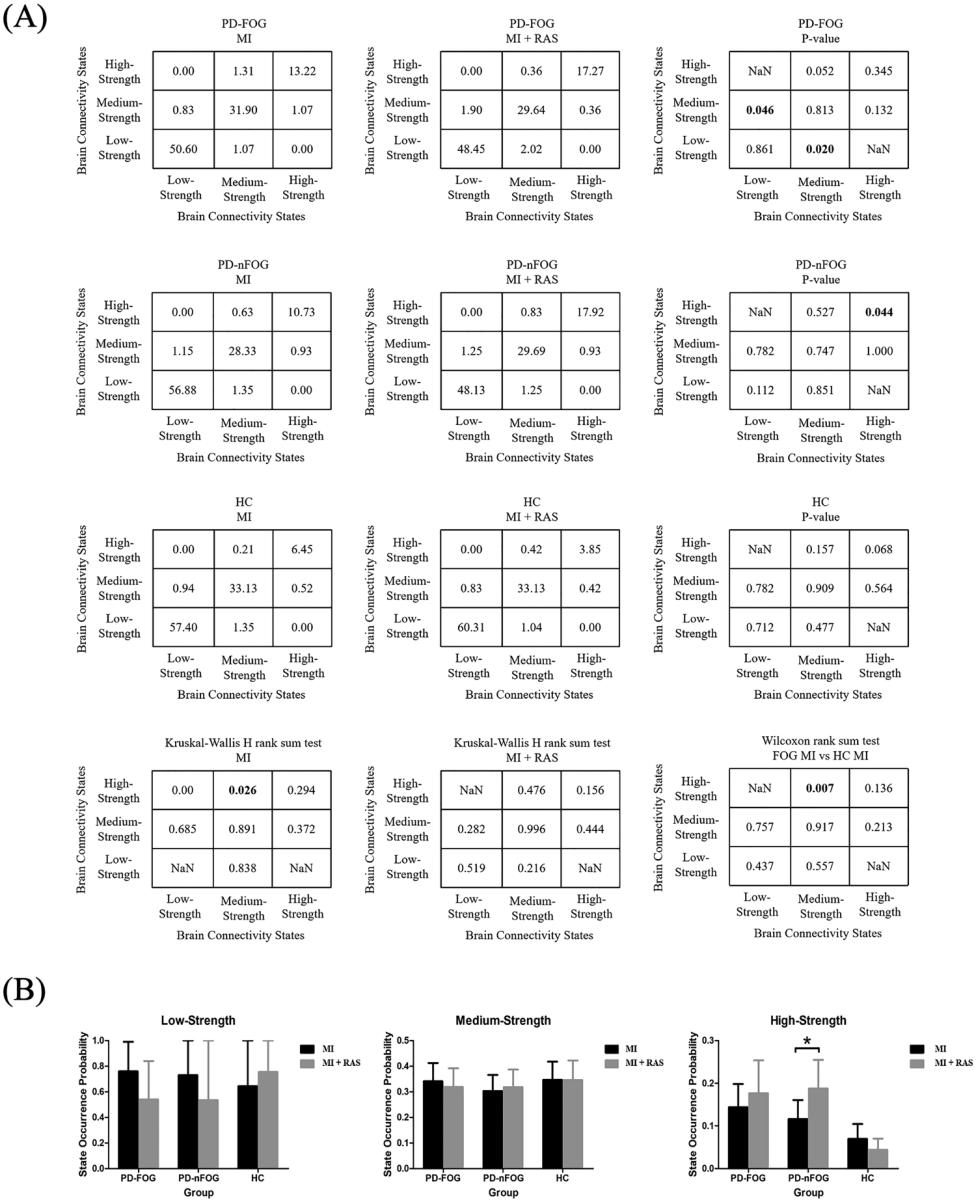
(A) State transition percentage. The value at (x, y) indicates the state transition percentage from state x to state y. Bold indicates p < 0.05. (B) State occurrence probability of low‐strength, medium‐strength and high‐strength brain connectivity states. MI, motor imagery; MI + RAS, motor imagery with rhythmic auditory stimulation. PD+FOG, Parkinson's disease patients with freezing of gait; PD‐FOG, Parkinson's disease patients without freezing of gait; HC, healthy controls. ∗ indicates p < 0.05.

The elbow method (Delgado et al. 2015), which detects the maximum perpendicular distance from the oblique line in Supplementary Material 2 Figure S1, was used to determine the optimal number of states. In our analysis, the optimal count was established as three across each cross‐validation round. K‐means clustering subsequently revealed three distinct brain connectivity states: low‐strength, medium‐strength, and high‐strength, each reflecting unique patterns of brain network connectivity, as illustrated in Figure 5. The high‐strength brain connectivity state displayed the greatest connectivity strength, represented by the most vivid colors, whereas the low‐strength brain connectivity state indicated the weakest connectivity, denoted by the lightest colors. To characterize these connectivity states, we calculated global efficiency and the clustering coefficient. Notably, the high‐strength brain connectivity state demonstrated significantly greater global efficiency and clustering coefficients than both the low‐strength and medium‐strength brain connectivity states, suggesting enhanced parallel information processing within the brain's functional networks (Supplementary Material 2 Figure S2). Figure 6A presents the state transition percentage. During imagined walking without RAS, PD+FOG patients exhibited a higher transition rate from the medium‐strength to the high‐strength brain connectivity state compared to healthy controls (p = 0.007). In contrast, during imagined walking with RAS, no significant differences were observed among the three groups. For PD+FOG patients, imagined walking with RAS led to a notable increase in transitions between low‐strength and medium‐strength brain connectivity states (p = 0.046, p = 0.020). Additionally, PD patients showed a significantly higher percentage of transitions within the high‐strength state under the RAS condition compared to the no‐RAS condition (p = 0.044). Figure 6B illustrates the state occurrence probability. During imagined walking with RAS, PD patients had a markedly higher likelihood of being in the high‐strength connectivity state compared to the no‐RAS condition (p = 0.037).

## 4 Discussion

Previous studies have demonstrated that RAS can improve gait impairment and FOG in PD patients, although the underlying mechanisms remain unclear. Our study identified alterations in cortical activation and dynamic brain functional connectivity in PD+FOG patients during an imagined walking task under RAS conditions, comparing these changes with PD‐FOG patients and HC. The main findings of our study are as follows: (1) PD+FOG patients exhibited significantly lower activation in the SMA and left M1‐LL during imagined walking without RAS. Additionally, they had a higher probability of transitioning to brain connectivity states characterized by high levels of information transfer compared to both PD‐FOG patients and HC. (2) Under the RAS condition, PD+FOG patients demonstrated notable activation in the SMA and left M1‐LL, accompanied by increased transitions between low‐ and medium‐strength brain connectivity states. These findings suggest that RAS may improve gait by modulating the activation of key motor regions and functional connectivity.

In our study, we utilized fNIRS to monitor cortical blood flow, which is closely linked to brain activity, in combination with MI to explore brain functions associated with FOG. FOG is a dynamic disorder that typically manifests during motion. While previous research on higher‐level neural activities related to FOG, such as those in the cerebellum, basal ganglia, locomotion regions, and cortex, has primarily stemmed from resting‐state fMRI studies (Nutt 2013, Gilat et al. 2015, Wang et al. 2016), capturing real‐time neural activity remains a challenge. To address this issue, the MI method proves to be a promising approach for investigating FOG‐related conditions (Huang et al. 2021). Visualizing a movement activates brain regions involved in planning and preparing for its execution, much like actually performing the movement (Hétu et al. 2013). For this reason, we selected the MI paradigm as a valuable tool in our study to explore brain functions associated with FOG.

To monitor real‐time neural activity, fNIRS was employed to capture measurements of oxygenated and deoxygenated hemoglobin concentrations, providing better temporal resolution compared to fMRI. One of the key advantages of fNIRS is its ability to directly reflect cortical activity, offering a relatively simple and cost‐effective alternative to fMRI, which requires large and expensive equipment, as well as more time‐consuming procedures. While fNIRS offers superior temporal resolution over fMRI, it still measures hemodynamic responses, which are slower than the direct electrical signals recorded by EEG. Therefore, while fNIRS can capture brain activity on a timescale of seconds, EEG would be more suitable for studies requiring even higher temporal resolution, on the order of milliseconds. This balance between temporal resolution and ease of implementation made fNIRS a favorable choice for our study, particularly given the focus on cortical brain activity in the context of FOG.

This study represents the first examination of activation changes in the frontal and parietal cortical areas in PD+FOG patients during an imagined walking task. Our results demonstrate that PD+FOG patients exhibited significantly reduced activation in both the SMA and the left M1‐LL compared to PD‐FOG patients and HC, particularly when performing the task without external RAS. These findings are consistent with prior fMRI research (Snijders et al. 2016, Li et al. 2018). The exact mechanisms underlying FOG remain unclear, with various hypotheses proposed; however, the SMA is widely considered a key node in these processes. The SMA plays a crucial role in connecting cognitive and motor neural resources (Nachev et al. 2008) and is an integral part of the basal ganglia‐cortical circuits, particularly involved in gait regulation, especially during the initiation of movement (Fling et al. 2014). A meta‐analysis has shown that SMA activation is reduced in PD patients compared to elderly controls, suggesting that decreased SMA activation may serve as a marker of impaired gait control (Gilat et al. 2019). Located posterior to the SMA, the left M1‐LL is the primary motor output area, responsible for transmitting motor commands via the corticospinal tract. It is essential for the accurate performance and regulation of lower limb movements (Takakusaki 2013). Previous research has shown that M1 excitability is reduced in PD patients both before and during freezing episodes, further highlighting the important role of M1 in FOG (Topka et al. 2022).

Interestingly, our study found that under RAS conditions, the diminished activity in the SMA and left M1‐LL in FOG patients was “rescued.” The prior clinical observations supported that RAS can improve gait parameters and reduce the risk of falls in PD and also can effectively decrease the frequency of FOG episodes and reduce turning time during both the ‘ON’ and ‘OFF’ medication phases (Trindade and Viana 2021, Thaut et al. 2019, Capato et al. 2020). The current findings provide neural mechanistic insights into RAS‐mediated gait improvements.

Our findings suggest that RAS may improve gait by engaging the SMA and M1‐LL. There is substantial evidence that the auditory and motor systems are interconnected at multiple levels, including cortical, subcortical, and spinal regions. The auditory system is particularly efficient at processing temporal information and links with motor brain structures, such as the basal ganglia, M1, SMA, and cerebellum, to synchronize rhythmic signals with motor responses (Thaut and Abiru 2010). It is believed that auditory rhythm serves as an external cue that synchronizes the motor system, reducing reliance on potentially impaired internal cues. This synchronization helps “entrain” motor patterns, facilitating smoother and more rhythmic movement. RAS is thought to support improved timing and consistency of gait, enhancing overall motor control. Compared to other external cueing methods like tactile or visual cues, RAS has shown the most pronounced benefits in gait training (Garzo et al. 2018). Even in the absence of overt movement, the cortical motor system remains active during the perception of auditory rhythms (Kung et al. 2013). Regular isochronous auditory stimuli can promote motor activation patterns by modulating cortical β oscillations or enhancing connectivity between the frontal, central‐parietal, and temporal regions (Koshimori and Thaut 2023).

The observed SMA and M1‐LL activation patterns align with the hierarchical organization of auditory‐motor integration. Research suggests that the SMA is connected to key nuclei within the auditory network, including those in the upper frontal, temporal, and parietal regions. The “perceptual dysfunction model” posits that the problem in visuo‐motor processing underlying FOG arises from inadequate transfer of visual input from the occipital regions to the somatosensory areas, and subsequently to the frontal regions responsible for generating appropriate motor plans. External rhythmic cues help compensate for degraded proprioceptive feedback by directly stimulating the remaining motor circuits. This external cueing mechanism synchronizes the motor system, reducing the reliance on potentially impaired internal signals (Bardakan et al. 2022). Auditory perception is also a key component of sensory processing. Upon exposure to rhythmic auditory stimuli, auditory‐motor coupling engages two primary sensory‐motor pathways: the auditory‐parieto‐cerebellar‐M1 pathway and the auditory‐striatal‐SMA pathway, collectively known as the Sensorimotor Theory of Rhythm Perception (SMT) (Lezama‐Espinosa and Hernandez‐Montiel 2020). Crucially, both pathways converge on the SMA‐M1 axis——a neural network particularly vulnerable in FOG pathophysiology.

Investigating brain dynamic functional connectivity is essential for elucidating the neural mechanisms that may underlie neurodegenerative diseases (Lu et al. 2023). Our study identified three distinct brain connectivity states—low‐, medium‐, and high‐strength—across PD+FOG patients, PD‐FOG patients, and HC. These states represent different degrees of information flow within the brain's functional networks. In the absence of RAS, PD+FOG patients exhibited a greater tendency to transition to states characterized by higher information transfer when imagining walking, compared to healthy controls. We speculate that this enhanced information transfer may act as a compensatory mechanism for the functional deficits experienced by FOG patients. Conversely, under RAS conditions, PD+FOG patients showed an increased frequency of transitions between low‐strength and medium‐strength brain connectivity states during the same imagined task. The number of transitions between states was quantified by counting the occurrences, where a higher number signifies lower stability of each state (Gan et al. 2023). Previous studies have shown that PD+FOG patients typically maintain a stable, monotonous ‘rigid’ state, while those without FOG display more dynamic switching in their information processing and communication capabilities. This discrepancy suggests that PD+FOG patients possess diminished abilities for dynamic functional compensation and regulation, hindering their ability to mobilize necessary resources and adapt to environmental demands in a timely manner (Gan et al. 2023). Our findings suggest that RAS can reduce the stability of brain connectivity states in PD+FOG patients, which may indicate enhanced dynamic regulatory capabilities under these conditions. This could represent one of the neural mechanisms through which RAS alleviates gait disturbances in these patients.

This study also has certain limitations. (1) Previous studies have shown that reduced SMA activation in PD patients can be improved through dopaminergic medication (Haslinger et al. 2001). Since this study was conducted during the patients' ON‐medication state, the influence of medication on SMA activation cannot be entirely ruled out. However, as all participants were in the “ON” medication state, this may help mitigate the potential impact of the medication to some extent. (2) The fNIRS device used in this study is limited to detecting signals from superficial cortical areas and cannot capture signals from deeper brain regions or the cerebellum. As a result, it is difficult to fully explore the impact of RAS on the entire brain network in PD patients with FOG. (3) Task‐related neuroimaging studies rely on precise measurement and control of task performance. The covert nature of imagined tasks poses challenges, as current methods do not definitively indicate whether subjects are genuinely engaging in the imagined tasks. The study did not obtain changes in gait parameters of subjects while walking under conditions with and without RAS, making it impossible to quantify the effectiveness of RAS behaviorally. Future research could utilize wearable inertial sensors to simultaneously collect near‐infrared signals and gait parameters, further clarifying the relationship among RAS, gait parameters, and brain function. (4) Our study incorporated a visual guide within the MI paradigm; however, it did not include a control condition that focused solely on visual input. Such a control condition would help to clarify whether the activation observed in gait‐related regions during MI is due to the imagined movement itself or simply the visual perception of stepping feet. In our study, all participants performed the motor imagery tasks under the same visual guidance, ensuring that the influence of visual input was consistent across all subjects. Furthermore, participants were explicitly instructed to focus on their own movements and engage in motor imagery, which reduces the likelihood that visual input alone was the primary driver of the observed brain activation patterns. Future studies that incorporate a control condition focusing exclusively on visual input could provide a more definitive understanding of the role of visual perception in brain activation during MI tasks. (5) Previous studies have found that the most effective frequencies for RAS in improving gait are typically within a 10% deviation above or below the subjects' preferred cadence (Harrison et al. 2019). However, in our study, due to the constraints of the music used, we provided all subjects with a standardized auditory cue frequency. This may have resulted in the auditory cue not being beneficial for the gait of all subjects, potentially affecting the interpretation of the study's results. Future research should consider tailoring the auditory cue frequency based on each subject's individual gait performance.

## 5 Conclusion

This study employed fNIRS to investigate alterations in brain region activation and functional connectivity in PD+FOG patients during an imagined walking task with and without RAS. Our findings indicate that RAS may improve gait impairments in FOG patients through two potential mechanisms: (1) activation of the SMA and the left M1‐LL; and (2) reduction of the stability of brain connectivity states in PD+FOG patients, which in turn may augment the dynamic regulatory capabilities of PD+FOG patients.

Furthermore, our study highlights the role of auditory perception as a crucial aspect of sensory processing in FOG, similar to visiomotor processing. We found that auditory stimuli, such as RAS, engage the SMA and M1 through auditory‐motor pathways during rhythm perception. These findings provide preliminary insights into the neural mechanisms underlying RAS's potential to alleviate FOG symptoms. This neural activation pattern offers a promising framework for understanding how RAS may serve as a therapeutic intervention, aiding clinicians in customizing RAS‐based neurofeedback therapies for PD+FOG patients.

## Author Contributions

**Chen Liu**: writing–original draft, investigation, data curation. **Miaomiao Hou**: methodology, software, formal analysis. **Shuili Yu**: data curation, investigation. **Ying Wan**: formal analysis, visualization, validation. **Zhenguo Liu**: funding acquisition, supervision, resources, conceptualization. **Jing Gan**: supervision, funding acquisition, project administration, resources, writing–review and editing, conceptualization.

## Conflicts of Interest

The authors declare no conflict of interest.

### Peer Review

The peer review history for this article is available at https://publons.com/publon/10.1002/brb3.70532

## Supporting information



Supporting Information

Supporting Information

Supporting Information

Figure S1 An example of the curve of within‐cluster sum of squares against number of clusters. The optimal state number was 3 during each round of cross validation.
Figure S2 Global efficiency and clustering coefficient for brain connectivity states. ∗ indicates p < 0.05.

## Data Availability

The data that support the findings of this study are available from the corresponding author upon reasonable request.

## References

[brb370532-bib-0001] Ashoori, A. , D. M. Eagleman , and J. Jankovic . 2015. “Effects of Auditory Rhythm and Music on Gait Disturbances in Parkinson's Disease.” Frontiers in Neurology 6: 234. 26617566 10.3389/fneur.2015.00234 PMC4641247

[brb370532-bib-0002] Bardakan, M. M. , G. R. Fink , L. Zapparoli , G. Bottini , E. Paulesu , and P. H. Weiss . 2022. “Imaging the Neural Underpinnings of Freezing of Gait in Parkinson's Disease.” NeuroImage: Clinical 35: 103123. 35917720 10.1016/j.nicl.2022.103123 PMC9421505

[brb370532-bib-0003] Capato, T. T. C. , N. M. de Vries , J. IntHout , et al. 2020. “Multimodal Balance Training Supported by Rhythmic Auditory Stimuli in Parkinson Disease: Effects in Freezers and Nonfreezers.” Physical Therapy 100, no. 11: 2023–2034. 32737973 10.1093/ptj/pzaa146 PMC7596891

[brb370532-bib-0004] Chen, J. L. , V. B. Penhune , and R. J. Zatorre . 2008. “Listening to Musical Rhythms Recruits Motor Regions of the Brain.” Cerebral Cortex 18, no. 12: 2844–2854. 18388350 10.1093/cercor/bhn042

[brb370532-bib-0005] Delgado, H. , X. Anguera , C. Fredouille , and J. Serrano . 2015. “Novel Clustering Selection Criterion for Fast Binary Key Speaker Diarization.” in Proceedings of the INTERSPEECH 2015, 16th Annual Conference of the International Speech Communication Association (pp. 3091–3095). ISCA.

[brb370532-bib-0006] Devlin, K. , J. T. Alshaikh , and A. Pantelyat . 2019. “Music Therapy and Music‐Based Interventions for Movement Disorders.” Current Neurology and Neuroscience Reports 19, no. 11: 83. 31720865 10.1007/s11910-019-1005-0

[brb370532-bib-0007] Dubois, B. , A. Slachevsky , I. Litvan , and B. Pillon . 2000. “The FAB: A Frontal Assessment Battery at Bedside.” Neurology 55, no. 11: 1621–1626. 11113214 10.1212/wnl.55.11.1621

[brb370532-bib-0008] Fling, B. W. , R. G. Cohen , M. Mancini , et al. 2014. “Functional Reorganization of the Locomotor Network in Parkinson Patients With Freezing of Gait.” PLoS ONE 9, no. 6: e100291. 24937008 10.1371/journal.pone.0100291 PMC4061081

[brb370532-bib-0009] Folstein, M. F. , S. E. Folstein , and P. R. McHugh . 1975. ““Mini‐Mental State”. A Practical Method for Grading the Cognitive State of Patients for the Clinician.” Journal of Psychiatric Research 12, no. 3: 189–198. 1202204 10.1016/0022-3956(75)90026-6

[brb370532-bib-0010] Gan, C. , M. Ji , H. Sun , et al. 2023. “Dynamic Functional Connectivity Reveals Hyper‐Connected Pattern and Abnormal Variability in Freezing of Gait of Parkinson's Disease.” Neurobiology of Disease 185: 106265. 37597816 10.1016/j.nbd.2023.106265

[brb370532-bib-0011] Gan, J. , W. Liu , X. Cao , et al. 2021. “Prevalence and Clinical Features of FOG in Chinese PD Patients, a Multicenter and Cross‐Sectional Clinical Study.” Frontiers in Neurology 12: 568841. 33763009 10.3389/fneur.2021.568841 PMC7982534

[brb370532-bib-0012] Garzo, A. , P. A. Silva , N. Garay‐Vitoria , et al. 2018. “Design and Development of a Gait Training System for Parkinson's Disease.” PLoS ONE 13, no. 11: e0207136. 30418993 10.1371/journal.pone.0207136 PMC6231661

[brb370532-bib-0013] Giladi, N. , and A. Nieuwboer . 2008. “Understanding and Treating Freezing of Gait in Parkinsonism, Proposed Working Definition, and Setting the Stage.” Movement Disorders 23: S423–S425. 18668629 10.1002/mds.21927

[brb370532-bib-0014] Gilat, M. , B. W. Dijkstra , N. D'Cruz , A. Nieuwboer , and S. J. G. Lewis . 2019. “Functional MRI to Study Gait Impairment in Parkinson's Disease: A Systematic Review and Exploratory ALE Meta‐Analysis.” Current Neurology and Neuroscience Reports 19, no. 8: 49. 31214901 10.1007/s11910-019-0967-2

[brb370532-bib-0015] Gilat, M. , J. M. Shine , C. C. Walton , C. O'Callaghan , J. M. Hall , and S. J. G. Lewis . 2015. “Brain Activation Underlying Turning in Parkinson's Disease Patients With and Without Freezing of Gait: A Virtual Reality fMRI Study.” NPJ Parkinson's Disease 1: 15020. 10.1038/npjparkd.2015.20 PMC5516618 28725687

[brb370532-bib-0016] Goetz, C. G. , B. C. Tilley , S. R. Shaftman , et al. 2008. “Movement Disorder Society‐Sponsored Revision of the Unified Parkinson's Disease Rating Scale (MDS‐UPDRS): Scale Presentation and Clinimetric Testing Results.” Movement Disorders 23, no. 15: 2129–2170. 19025984 10.1002/mds.22340

[brb370532-bib-0017] Grahn, J. A. , and M. Brett . 2007. “Rhythm and Beat Perception in Motor Areas of the Brain.” Journal of Cognitive Neuroscience 19, no. 5: 893–906. 17488212 10.1162/jocn.2007.19.5.893

[brb370532-bib-0018] Harrison, E. C. , A. P. Horin , and G. M. Earhart . 2019. “Mental Singing Reduces Gait Variability More Than Music Listening for Healthy Older Adults and People with Parkinson Disease.” Journal of Neurologic Physical Therapy 43, no. 4: 204–211. 31449178 10.1097/NPT.0000000000000288 PMC6744333

[brb370532-bib-0019] Haslinger, B. , P. Erhard , N. Kämpfe , et al. 2001. “Event‐Related Functional Magnetic Resonance Imaging in Parkinson's Disease Before and After Levodopa.” Brain 124, no. 3: 558–570. 11222456 10.1093/brain/124.3.558

[brb370532-bib-0020] Hétu, S. , M. Grégoire , A. Saimpont , et al. 2013. “The Neural Network of Motor Imagery: An ALE Meta‐Analysis.” Neuroscience and Biobehavioral Reviews 37, no. 5: 930–949. 23583615 10.1016/j.neubiorev.2013.03.017

[brb370532-bib-0021] Hou, X. , Z. Zhang , C. Zhao , et al. 2021. “NIRS‐KIT: A MATLAB Toolbox for Both Resting‐State and Task fNIRS Data Analysis.” Neurophotonics 8, no. 1: 010802. 33506071 10.1117/1.NPh.8.1.010802 PMC7829673

[brb370532-bib-0022] Huang, H. C. , C. M. Chen , M. K. Lu , et al. 2021. “Gait‐Related Brain Activation during Motor Imagery of Complex and Simple Ambulation in Parkinson's Disease with Freezing of Gait.” Frontiers in aging neuroscience 13: 731332. 34630069 10.3389/fnagi.2021.731332 PMC8492994

[brb370532-bib-0023] Huo, C. , G. Xu , H. Xie , et al. 2024. “Functional Near‐Infrared Spectroscopy in Non‐Invasive Neuromodulation.” Neural Regeneration Research 19, no. 7: 1517–1522. 38051894 10.4103/1673-5374.387970 PMC10883499

[brb370532-bib-0024] Koshimori, Y. , and M. H. Thaut . 2023. “Rhythmic Auditory Stimulation as a Potential Neuromodulator for Parkinson's Disease.” Parkinsonism & Related Disorders 113: 105459. 37277293 10.1016/j.parkreldis.2023.105459

[brb370532-bib-0025] Kung, S. J. , J. L. Chen , R. J. Zatorre , and V. B. Penhune . 2013. “Interacting Cortical and Basal Ganglia Networks Underlying Finding and Tapping to the Musical Beat.” Journal of Cognitive Neuroscience 25, no. 3: 401–420. 23163420 10.1162/jocn_a_00325

[brb370532-bib-0026] Lezama‐Espinosa, C. , and H. L. Hernandez‐Montiel . 2020. “Neuroscience of the Auditory‐Motor System: How Does Sound Interact With Movement?” Behavioural Brain Research 384: 112535. 32044405 10.1016/j.bbr.2020.112535

[brb370532-bib-0027] Li, J. , Y. Yuan , M. Wang , et al. 2018. “Decreased Interhemispheric Homotopic Connectivity in Parkinson's Disease Patients With Freezing of Gait: A Resting State fMRI Study.” Parkinsonism & Related Disorders 52: 30–36. 29602542 10.1016/j.parkreldis.2018.03.015

[brb370532-bib-0028] Lu, J. , X. Zhang , Y. Wang , et al. 2023. “An fNIRS‐Based Dynamic Functional Connectivity Analysis Method to Signify Functional Neurodegeneration of Parkinson's Disease.” Ieee Transactions on Neural Systems and Rehabilitation Engineering 31: 1199–1207. 37022412 10.1109/TNSRE.2023.3242263

[brb370532-bib-0029] Mirelman, A. , P. Bonato , R. Camicioli , et al. 2019. “Gait Impairments in Parkinson's Disease.” Lancet Neurology 18, no. 7: 697–708. 30975519 10.1016/S1474-4422(19)30044-4

[brb370532-bib-0030] Nachev, P. , C. Kennard , and M. Husain . 2008. “Functional Role of the Supplementary and Pre‐Supplementary Motor Areas.” Nature Reviews Neuroscience 9, no. 11: 856–869. 18843271 10.1038/nrn2478

[brb370532-bib-0031] Nieuwboer, A. , L. Rochester , T. Herman , et al. 2009. “Reliability of the New Freezing of Gait Questionnaire: Agreement Between Patients With Parkinson's Disease and Their Carers.” Gait & Posture 30, no. 4: 459–463. 19660949 10.1016/j.gaitpost.2009.07.108

[brb370532-bib-0032] Nutt, J. G. 2013. “Higher‐Level Gait Disorders: An Open Frontier.” Movement Disorders 28, no. 11: 1560–1565. 24132844 10.1002/mds.25673

[brb370532-bib-0033] Postuma, R. B. , D. Berg , M. Stern , et al. 2015. “MDS Clinical Diagnostic Criteria for Parkinson's Disease.” Movement Disorders 30, no. 12: 1591–1601. 26474316 10.1002/mds.26424

[brb370532-bib-0034] Raglio, A. 2015. “Music Therapy Interventions in Parkinson's Disease: The State‐of‐the‐Art.” Front Neurol 6: 185. 26379619 10.3389/fneur.2015.00185 PMC4553388

[brb370532-bib-0035] Rossetti, H. C. , L. H. Lacritz , C. M. Cullum , and M. F. Weiner . 2011. “Normative Data for the Montreal Cognitive Assessment (MoCA) in a Population‐Based Sample.” Neurology 77, no. 13: 1272–1275. 21917776 10.1212/WNL.0b013e318230208a

[brb370532-bib-0036] Snijders, A. H. , K. Takakusaki , B. Debu , et al. 2016. “Physiology of Freezing of Gait.” Annals of Neurology 80, no. 5: 644–659. 27649270 10.1002/ana.24778

[brb370532-bib-0037] Takakusaki, K. 2013. “Neurophysiology of Gait: From the Spinal Cord to the Frontal Lobe.” Movement Disorders 28, no. 11: 1483–1491. 24132836 10.1002/mds.25669

[brb370532-bib-0038] Thaut, M. H. , and M. Abiru . 2010. “Rhythmic Auditory Stimulation in Rehabilitation of Movement Disorders: A Review Of Rhythmic Auditory Stimulation in Rehabilitation 263.” Source: Music Perception An Interdisciplinary Journal Music Perception 27: 263–269.

[brb370532-bib-0039] Thaut, M. H. , R. R. Rice , T. Braun Janzen , C. P. Hurt‐Thaut , and G. C. McIntosh . 2019. “Rhythmic Auditory Stimulation for Reduction of Falls in Parkinson's Disease: a Randomized Controlled Study.” Clinical Rehabilitation 33, no. 1: 34–43. 30033755 10.1177/0269215518788615

[brb370532-bib-0040] Tomlinson, C. L. , R. Stowe , S. Patel , C. Rick , R. Gray , and C. E. Clarke . 2010. “Systematic Review of levodopa Dose Equivalency Reporting in Parkinson's Disease.” Movement Disorders 25, no. 15: 2649–2653. 21069833 10.1002/mds.23429

[brb370532-bib-0041] Topka, M. , M. Schneider , C. Zrenner , P. Belardinelli , U. Ziemann , and D. Weiss . 2022. “Motor Cortex Excitability is Reduced During Freezing of Upper Limb Movement in Parkinson's disease.” NPJ Parkinsons Dis 8, no. 1: 161. 36424411 10.1038/s41531-022-00420-w PMC9691624

[brb370532-bib-0042] Trindade, M. F. D. , and R. A. Viana . 2021. “Effects of Auditory or Visual Stimuli on Gait in Parkinsonic Patients: A Systematic Review.” Porto Biomed J 6, no. 4: e140. 34368491 10.1097/j.pbj.0000000000000140 PMC8341369

[brb370532-bib-0043] Wang, M. , S. Jiang , Y. Yuan , et al. 2016. “Alterations of Functional and Structural Connectivity of Freezing of Gait in Parkinson's Disease.” Journal of Neurology 263, no. 8: 1583–1592. 27230857 10.1007/s00415-016-8174-4

[brb370532-bib-0044] Xia, M. , J. Wang , and Y. He . 2013. “BrainNet Viewer: A Network Visualization Tool for Human Brain Connectomics.” PLoS ONE 8, no. 7: e68910. 23861951 10.1371/journal.pone.0068910 PMC3701683

