# Supplementary figures and images for: An Exploratory Analysis of Rhythmic Auditory Stimulation's Impact on Brain Function in Parkinson's Disease Patients With Freezing of Gait
Source: Brain Behav. 2025 May 8;15(5):e70532. doi: 10.1002/brb3.70532 (PMC12060216; doi:10.1002/brb3.70532)

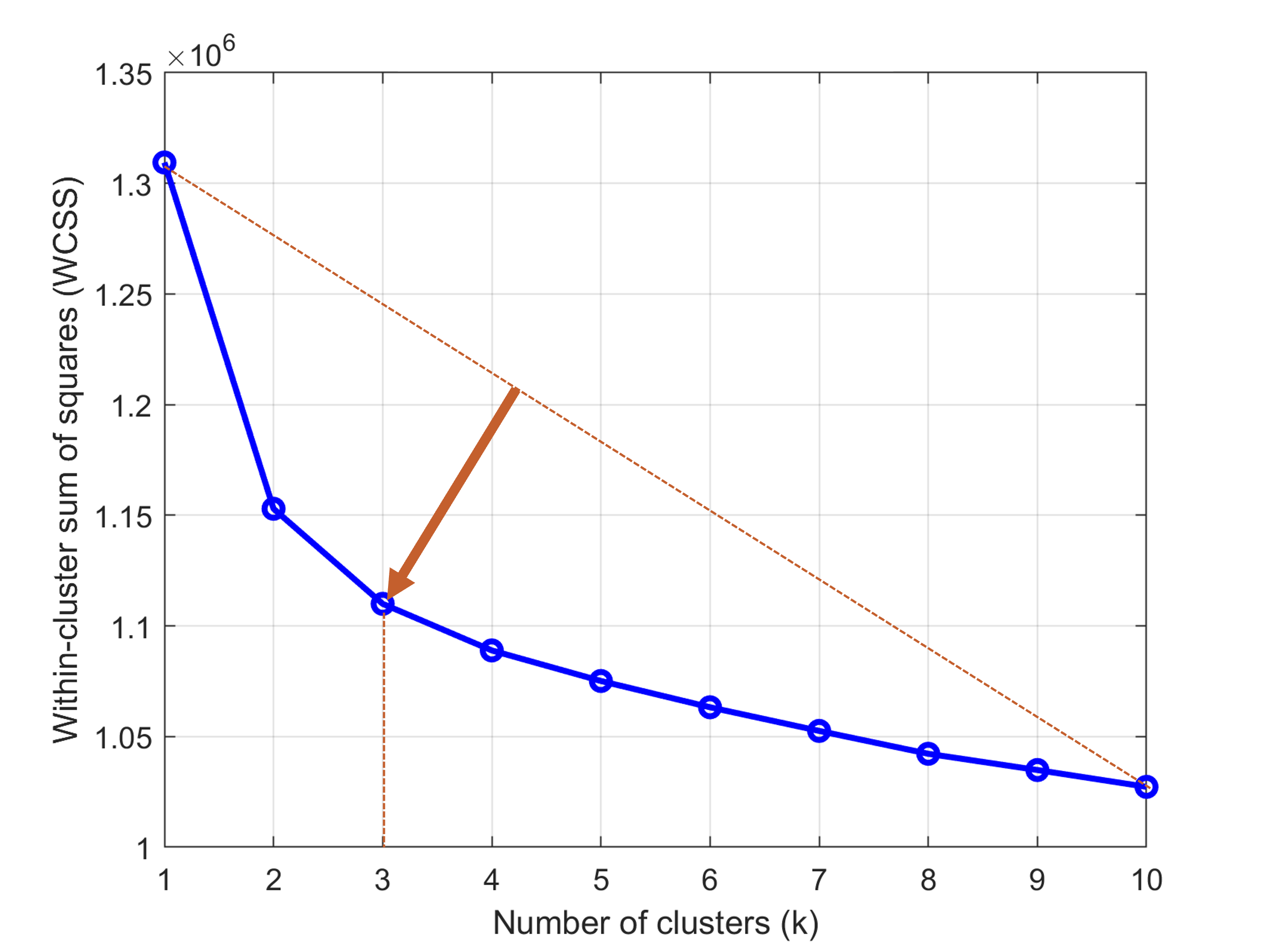

Supplement: Supplementary file 1 — Supporting Information [file BRB3-15-e70532-s004.png]

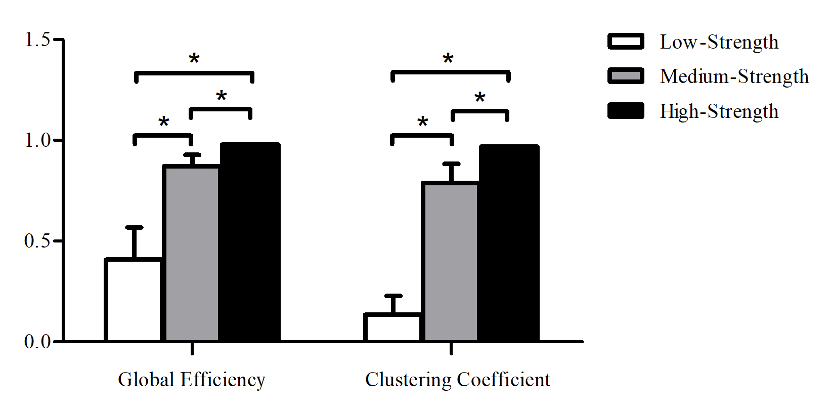

Supplement: Supplementary file 2 — Supporting Information [file BRB3-15-e70532-s001.png]
