# Supplementary material for: An Exploratory Analysis of Rhythmic Auditory Stimulation's Impact on Brain Function in Parkinson's Disease Patients With Freezing of Gait
Source: Brain Behav. 2025 May 8;15(5):e70532. doi: 10.1002/brb3.70532 (PMC12060216; doi:10.1002/brb3.70532)
Supplement: Supplementary file 3 — Supporting Information [file BRB3-15-e70532-s003.docx]

**Supplementary methods:**

**General linear model (GLM) Operation Process:**

**1. Task Design and Stimulus Event Modeling**

In fNIRS experiments, different stimulus events (e.g., motor imagery with or without RAS) are presented to participants at specific time intervals. These events are treated as independent variables in GLM analysis, with the design matrix capturing the timing, duration, and type of each stimulus. The design matrix records the presentation of stimuli at different time points, where each column represents a specific task condition or stimulus, and each row corresponds to a time point. The matrix values are typically binary (0 or 1), indicating the presence or absence of a particular stimulus.

**2. GLM Modeling**

The GLM assumes a linear relationship between brain activity (e.g., oxygenated hemoglobin concentration) and stimulus events. The general form of the GLM is:

[ Y = X\beta + \epsilon]

Where:

- (Y) is the observed signal (e.g., oxygenated hemoglobin time series),

- (X) is the design matrix (timing and conditions of stimuli),

- (\beta) is the vector of regression coefficients, representing the strength and direction of the relationship between each task condition and brain activity,

- (\epsilon) is the residual error term, capturing noise and unmodeled variability.

**3. Beta Value Calculation**

The beta values are computed using least squares estimation, which minimizes the difference between predicted and actual values. Positive beta values indicate an increase in brain activity, while negative beta values indicate a decrease.
