# Supplementary material for: An Exploratory Analysis of Rhythmic Auditory Stimulation's Impact on Brain Function in Parkinson's Disease Patients With Freezing of Gait
Source: Brain Behav. 2025 May 8;15(5):e70532. doi: 10.1002/brb3.70532 (PMC12060216; doi:10.1002/brb3.70532)
Supplement: Supplementary file 4 — Figure S1 An example of the curve of within‐cluster sum of squares against number of clusters. The optimal state number was 3 during each round of cross validation. Figure S2 Global efficiency and clustering coefficient for brain connectivity states. ∗ indicates p < 0.05. [file BRB3-15-e70532-s002.docx]

**Supplementary Figure Legends:**


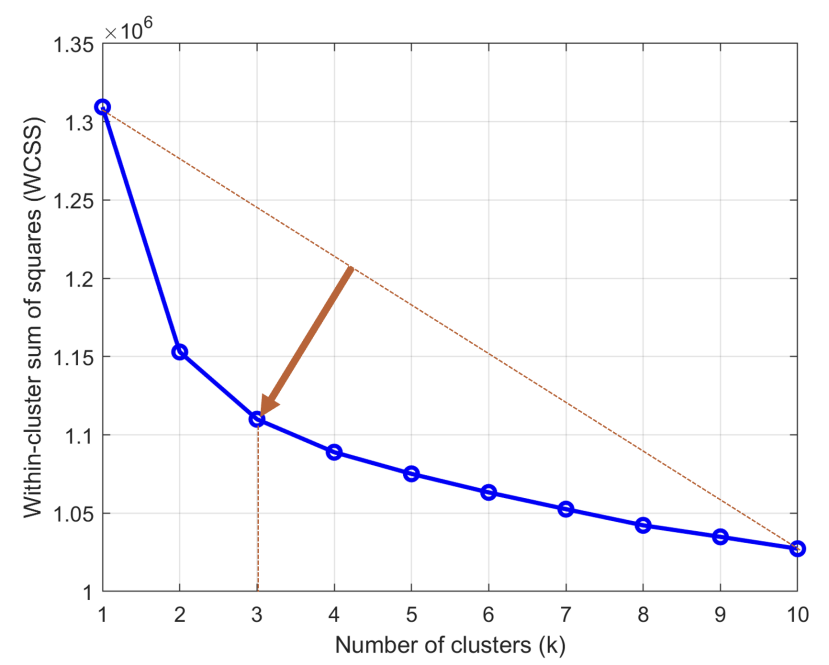


Figure S1 An example of the curve of within-cluster sum of squares against number of clusters. The optimal state number was 3 during each round of cross validation.


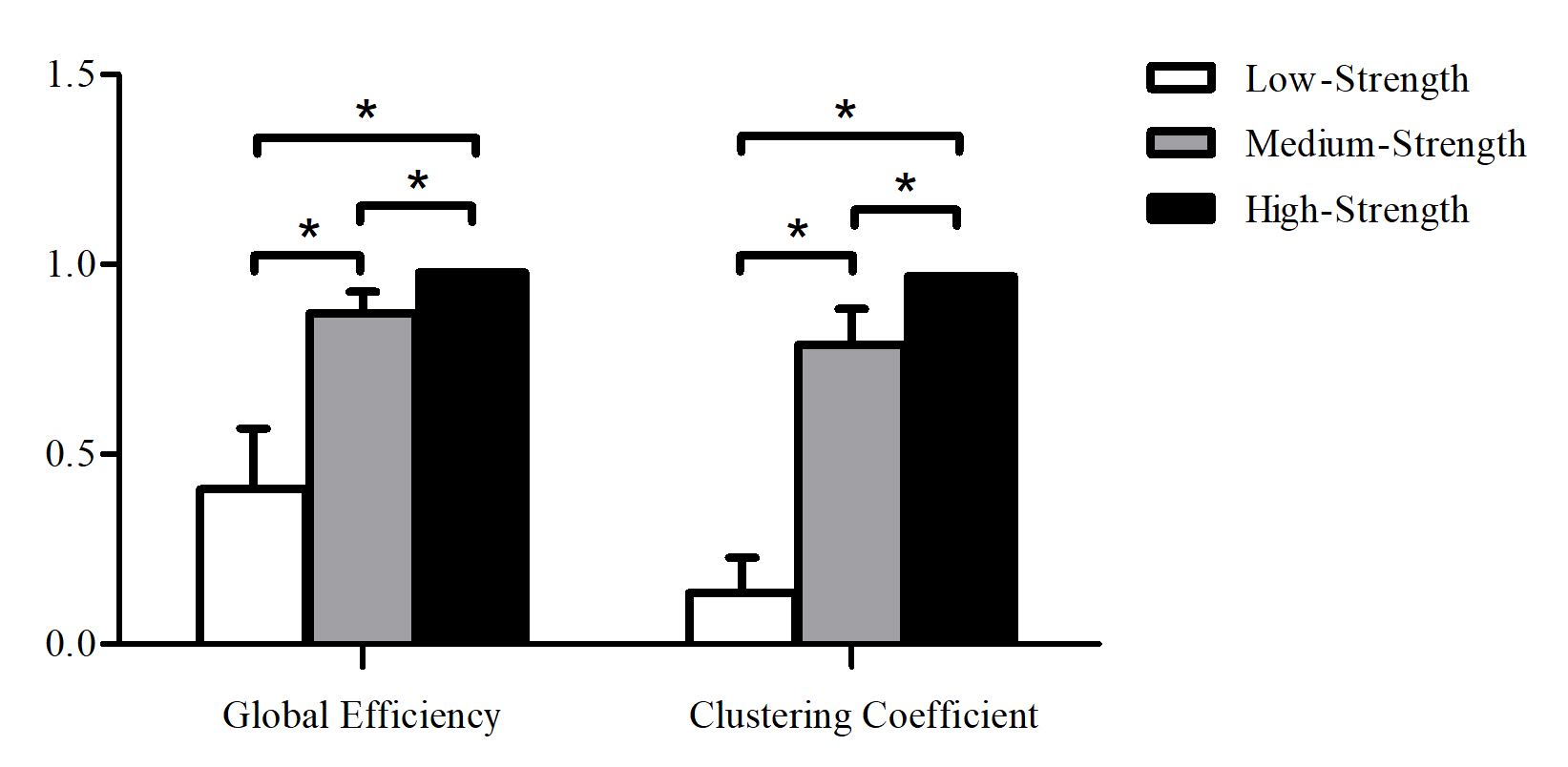


Figure S2 Global efficiency and clustering coefficient for brain connectivity states. ∗ indicates p < 0.05.
